# A new nonlinear method for calculating growing degree days

**DOI:** 10.1038/s41598-018-28392-z

**Published:** 2018-07-05

**Authors:** Guanglin Zhou, Quanjiu Wang

**Affiliations:** 1 Institute of Water Resources and Hydro-electric Engineering, Xi’an University of Technology, Xi’an, 710048 China; 2 State Key Laboratory of Eco-hydraulics in Northwest Arid Region of China, Xi’an University of Technology, Xi’an, 710048 China

## Abstract

Precise calculations of growing degree days (GDD) are an important component in crop simulation models and managerial decisions. Traditional methods for calculating GDD assume linear developmental responses to temperature and cannot precisely account for the delay in growth or development at temperatures above the optimal temperature (T_opt_). A new nonlinear method for calculating GDD was developed. Variations in the prediction of the dates since sowing to various developmental stages and performance measures for describing the accumulation of dry matter by GDD for two widely planted crops (corn and wheat) were used to evaluate the new method in comparison with the traditional methods. The new method predicted the dates of the developmental stages more precisely (date variations reduced by 1 d), and the errors for the predictions of the accumulation of dry matter for winter wheat and corn were smaller. The method was most promising for spring wheat. The new method was more stable and more precise than traditional methods, especially when T_opt_ was lower than the maximum air temperature.

## Introduction

Precise calculations of growing degree days (GDD) are important in models simulating crop growth and for the management of field crops. GDD is also a climatic feature. The use of GDD has vastly improved the description and prediction of phenological events compared with other approaches, such as time of year or number of days, particularly for crop phenology and developmental stage^1,2^.

The relationship between the rate of development and temperature is key for calculating GDD. A linear relationship, which assumes that the rate is proportional to the temperature above a threshold, is used most widely and is often precise for intermediate temperatures^3–7^. However, the assumption of rate-temperature linearity will yield errors when temperatures tend toward extremes under variable conditions^8,9^, i.e., a linear relationship between temperature and plant growth is inappropriate in long-term studies, especially for complete life cycles^10^. Many methods that assume a nonlinear relationship have thus been developed, each with strengths and weaknesses. For example, a bilinear approach has been adopted^11,12^ in which the responses to sub- and supra-optimal temperatures are described by different linear equations, and the real response curve is generally smooth. An exponential equation is usually effective for simulating responses at low to intermediate temperatures but not for simulating responses to high temperatures because it does not allow for a rate of development at high temperatures^13^. A quadratic equation is a simple model that can allow a lower rate of development at high temperatures^14,15^. However, the temperature response is rarely a symmetric parabola, and applications of quadratic models may thus be inaccurate. Yin adopted a beta function containing three parameters (the cardinal temperatures) to describe the temperature response and reported successful simulations of the development of several crops (corn, wheat, barley, sorghum, and beans); the method was superior to widely used thermal time approaches in predicting crop developmental stages^16,17^. The approach accounts for the asymmetric temperature response for the developmental rate and the decline in the rate above T_opt_.

Traditional GDD correlates developmental rate linearly to temperatures above the lower threshold temperature in some applications of the procedure; however, linearization is often criticized for its oversimplification despite being widely used^18^. A more stable and less variable GDD, which should be calculated by a precise method, is needed so that the stages of the crop growth period may be accurately compared and predicted regardless of environmental conditions. However, traditional approaches cannot accurately account for the delay in growth or development at temperatures above the optimal temperature (T_opt_). The nonlinear relationships between developmental rate and temperature discussed above are rarely applied to calculate GDD. Therefore, the objective of this study was to develop an improved nonlinear method of calculating GDD and to evaluate the accuracy and applicability of the method by comparison with other methods.

## Materials and Methods

GDD, which is cumulative daily thermal time (DTT), is calculated as:

1 $$GDD=\sum DTT$$

Calculating DTT is key to the methods for calculating GDD, and present methods for calculating GDD differ in their method for calculating DTT.

### Present widely used methods

McMaster and Wilhelm (1997) proposed two methods for calculating DTT (Methods 1 and 2) needed for calculating GDD, and both methods have been widely used in recent studies^18–22^. Method 1, which is simpler than Method 2, calculates DTT as:

2 $$DTT=\{\begin{array}{ll}0 & {T}_{avg} < {T}_{b}\\ {T}_{avg}-{T}_{b} & {T}_{b} < {T}_{avg} < {T}_{u}\\ {T}_{u}-{T}_{b} & {T}_{avg} > {T}_{u}\end{array}$$

where T_max_ is the maximum temperature, T_min_ is the minimum temperature, T_avg_ = (T_max_ + T_min_)/2, T_b_ is the base temperature, and T_u_ is the upper threshold temperature.

Method 2 is an improvement on Method 1. T_b_ is compared with T_u_ before the average temperature (T_avg_′) is calculated. T_m_ and T_n_ are adjusted if they are <T_b_ or >T_u_. In this method, DTT is given by:

3 $$DTT=\{\begin{array}{ll}0 & \,{T}_{avg} < {T}_{b}\\ {T}_{avg}\text{'}-{T}_{b} & {T}_{b} < {T}_{avg} < {T}_{u}\\ {T}_{u}-{T}_{b} & \,{T}_{u} < {T}_{avg}\end{array}$$

where T_m_ = min (T_max_, T_u_), T_n_ = max (T_m_, T_b_), and T_avg_′ = (T_m_ + T_n_)/2.

Method 3 introduces T_opt_. Thermal time increases linearly until T_opt_ is reached and decreases rapidly by another linear relationship at supraoptimal temperatures (T > T_opt_)^9,11,23^. Method 3 is calculated as:

4 $$HTT=\{\begin{array}{ll}0 & {T}_{h} < {T}_{b}\\ {T}_{h}-T & {T}_{b}\le {T}_{h}\le {T}_{opt}\\ \frac{{T}_{opt}-{T}_{b}}{{T}_{u}-{T}_{opt}}({T}_{u}-{T}_{h}) & {T}_{opt} < {T}_{h}\le {T}_{u}\\ 0 & {T}_{u} < {T}_{h}\end{array}$$

5 $$DTT=(\sum _{1}^{24}HT{T}_{i})/24$$

where HTT is hourly thermal time and T_h_ is the hourly temperature.

However, method 3 is linear both above and below a sharp T_opt_, and the value of thermal time may thus fluctuate near T_opt_. The response curve is also less smooth.

### Description of the new method

The beta-distribution function has a density of zero when x ≤ 0 or ≥1 and a maximum density at an optimal x between 0 and 1. Replacing the dependent variable x with temperature T between T_b_ and T_u_ leads to the following expression that can be used to describe the relationship between developmental rate and temperature^24^:

6 $$r={R}_{{\rm{\max }}}\{\begin{array}{ll}0 & {T}_{h} < {T}_{b}\\ (\frac{{T}_{h}-{T}_{b}}{{T}_{opt}-{T}_{b}}){(\frac{{T}_{u}-{T}_{h}}{{T}_{u}-{T}_{opt}})}^{\frac{{T}_{u}-{T}_{opt}}{{T}_{opt}-{T}_{b}}} & {T}_{b}\le {T}_{h}\le {T}_{u}\\ 0 & {T}_{u} < {T}_{h}\end{array}$$

where R_max_ is the maximum developmental rate and T_h_ is the hourly temperature.

The equation assumes that the developmental rate of the crop is maximal at T_opt_^11,25^ because the contribution of thermal time is T_opt_−T_b_ °C. The hourly thermal time can then be calculated by a beta-distribution function method (BFM) as:

7 $$HTT=\{\begin{array}{ll}0 & {T}_{h} < {T}_{b}\\ (\frac{{T}_{h}-{T}_{b}}{{T}_{opt}-{T}_{b}}){(\frac{{T}_{u}-{T}_{h}}{{T}_{u}-{T}_{opt}})}^{\frac{{T}_{u}-{T}_{opt}}{{T}_{opt}-{T}_{b}}}({T}_{opt}-{T}_{b}) & {T}_{b}\le {T}_{h}\le {T}_{u}\\ 0 & {T}_{u} < {T}_{h}\end{array}$$

The equation has three parameters (T_b_, T_opt_, and T_u_), and the thermal time will be zero if T = T_b_ or if T = T_u_ and will be maximum if T = T_opt_.

**Figure 1:**
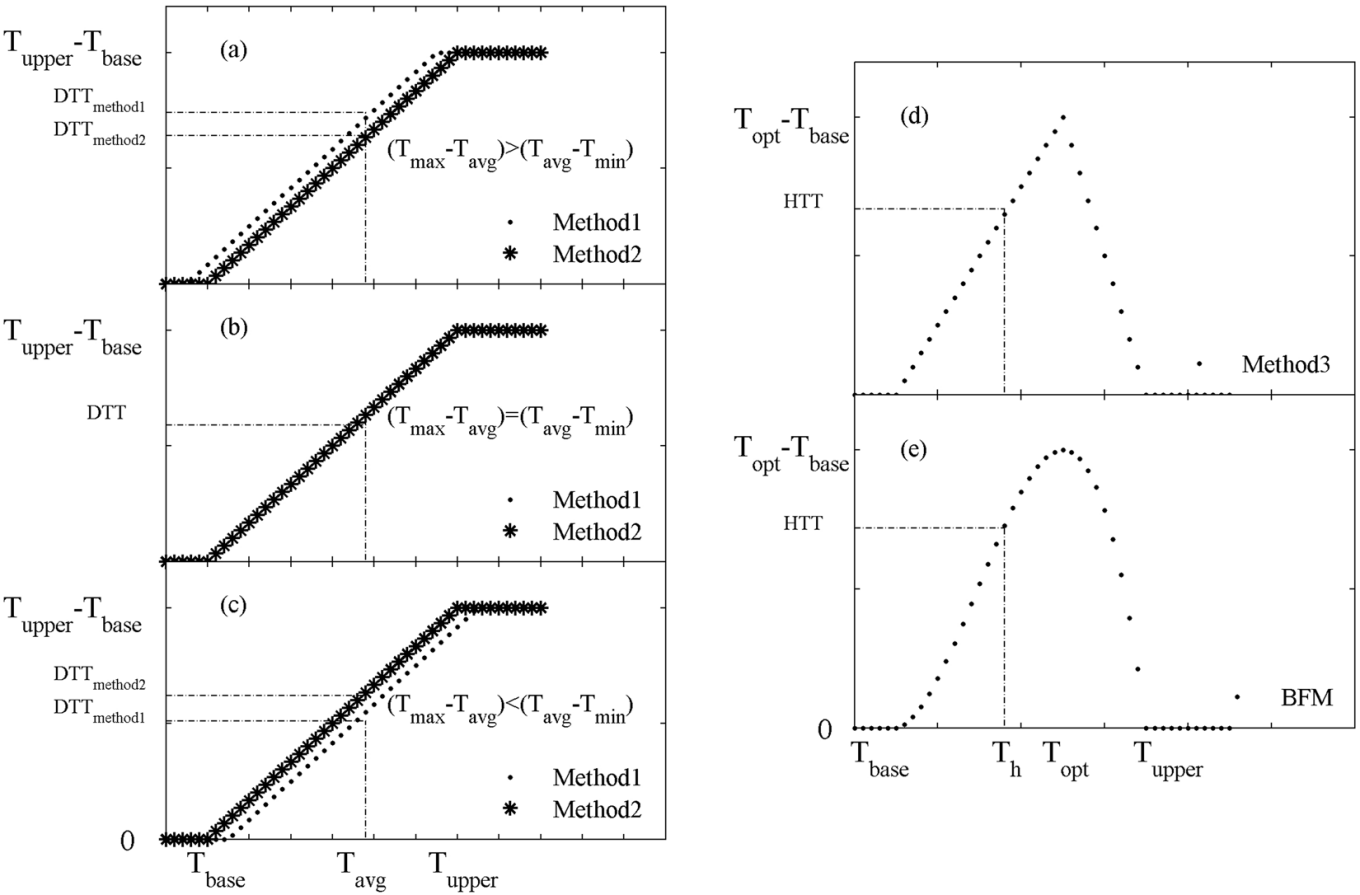
Comparison of the four methods used to calculate thermal time (DTT and HTT).

Figure 1 shows the comparison of the four methods used to calculate DTT and HTT. The daily GDD of Methods 1 and 2 is the same when (T_max_ − T_avg_) = (T_avg_ − T_min_), the daily GDD is higher for Method 1 than that for Method 2 when (T_max_ − T_avg_) > (T_avg_ − T_min_), and the daily GDD is lower for Method 1 than that for Method 2 when (T_max_ − T_avg_) < (T_avg_ − T_min_). The two methods involve a linearly-increasing segment with temperature T up to T_u_, beyond which GDD = T_u_ − T_b_. The curve of the temperature response for BFM is smoother than that for Method 3.

### Model evaluation

GDD is frequently used to describe biological processes but no canonical forms are available for calculating GDD. Therefore, we used the coefficient of variation (CV) of predictions of developmental stages and the performance of the accumulation of dry matter described by GDD to evaluate the four methods.

### Predicting developmental stages

The observed dates of developmental stages from field data were used to calculate the GDD required to reach a particular stage by the four methods. However, the GDD required to reach a particular stage calculated by a particular method with observed dates in different years was not always the same.

The CV of the dates predicted by one method for calculating GDD was used to test the performance of the method. The lower the CV, the better the prediction. CV was calculated as:

8 $$CV=\frac{S{D}_{GDD}}{GD{D}_{m}}$$

where *SD*_*GDD*_ is the standard derivation of the annual GDD required for a particular developmental stage since sowing, and *GDD*_*m*_ is the mean annual daily GDD during the developmental stage since sowing.

Willomtt^26^ proposed a refined index of agreement (d_r_) for evaluating model performance, defined as:

9 $${d}_{r}=\{\begin{array}{ll}1-\frac{\sum _{i=1}^{n}|{P}_{i}-{O}_{i}|}{2\sum _{i=1}^{n}|{O}_{i}-\bar{O}|} & \sum _{i=1}^{n}|{P}_{i}-{O}_{i}|\le c\sum _{i=1}^{n}|{O}_{i}-\bar{O}|\\ \frac{2\sum _{i=1}^{n}|{O}_{i}-\bar{O}|}{\sum _{i=1}^{n}|{P}_{i}-{O}_{i}|}-1 & \sum _{i=1}^{n}|{P}_{i}-{O}_{i}| > c\sum _{i=1}^{n}|{O}_{i}-\bar{O}|\end{array}$$

where *O*_*i*_ represents the developmental stage of an observation, *P*_*i*_ represents the method of prediction of the developmental stage, and $\bar{O}$ represents the mean value of an observation. A d_r_ of 1 indicates perfect agreement between prediction and observation.

### Describing the accumulation of dry matter

The accumulation of dry matter is commonly described by a logistic model as a function of GDD^19,27–30^. The normalized logistic model was fitted by GDD to test the performance of the methods for calculating GDD as follows:

10 $$y=\frac{{Y}_{{\rm{o}}}}{{Y}_{{\rm{m}}}}=\frac{1}{1+{e}^{a+bGDD}}$$

where *Y*_*o*_ is the observed amount of dry matter, *Y*_*m*_ is the maximum amount of dry matter, *y* is the normalized amount of dry matter, and *a* and *b* are coefficients.

The fitted amount of dry matter for each location was evaluated by the root mean square error (RMSE), defined as:

11 $$R{\rm{M}}SE=\sqrt{\frac{{\sum ({y}_{fi}-{y}_{oi})}^{2}}{n}}$$

where *y*_*oi*_ and *y*_*fi*_ are the observed and fitted amounts of dry matter, respectively, and n is the number of observations.

### Data for calculations

#### Crop phenological data

Data for wheat (a C3 crop) and corn (a C4 crop), which are widely planted around the world in a large range of cardinal temperatures (T_b_, T_opt_, and T_u_), were used in this study.

T_b_, T_opt_, and T_u_ for corn were 8, 33, and 40 °C, respectively^7,11,31,32^. Spring and winter wheat respond differently to temperature, and the three cardinal temperatures for the two wheat crops were thus not identical in the study^33^. T_b_, T_opt_, and T_u_ for winter wheat were 0, 24, and 45 °C for the vegetative phase (from emergence to heading)^7,31^ and 8, 29, and 40 °C for the reproductive phase (from heading to maturity), respectively^34–36^. T_b_, T_opt_, and T_u_ for spring wheat were 0, 24, and 42 °C, respectively^7,37–41^.

The phenological and temperature data for the two crops were obtained from agro-meteorological experimental stations maintained by the Chinese Meteorological Administration. The records for both crop phenologies were available from 1991 to 2010, although some records were missing. Data from the stations with records for more than eight years were used for our analysis (Table 1). The five stations (Turpan, Korla, Hezhang, Xinxiang, and Xianyou) represent a wide range of climatic conditions for the two crops. For example, Turpan station is in the warm-temperate and continental-drought climatic zone in northwestern China, which has extreme aridity, high temperatures, and a large temperature difference during the growth period. In contrast, Xianyou station is in a subtropical-monsoon climatic zone in southeastern China.

**Table 1:** Descriptions of locations used for the phenological analyses.

Location and length of record	Latitude (°)	Longitude (°)	Elevation (m)	Mean annual daily maximum temperature (°C)	Mean annual daily minimum temperature (°C)	Mean annual precipitation (mm)	Climate	Crop
Turpan (1993–2001)	42.56	89.12	35	21.6	8.0	16.0	Temperate continental climate	Spring wheat
Korla (2002–2009)	41.45	86.08	932	18.7	6.8	58.1	Temperate continental climate	Corn
Hezhang (1996–2004)	27.13	104.71	1996	17.9	9.6	926.7	Temperate monsoon climate	Winter wheat and corn
Xinxiang (2002–2009)	35.30	113.93	70	19.6	10.4	656.3	Temperate monsoon climate	Corn
Xianyou (1992–2005)	25.36	118.70	60	24.8	17.8	1610.2	Subtropical monsoon climate	Winter wheat

Source of data: Chinese Meteorological Administration (CMA) http://data.cma.cn.

### Data for total accumulated aboveground dry matter

Data for the total accumulated aboveground dry matter from two locations where the two crops are widely planted and have different climates (northern Xinjiang and central Shaanxi Plain) were reported in previous studies and were used to test the performance of the methods (Table 2)^42–51^. Northern Xinjiang has a temperate continental climate with a mean annual temperature of 8.1 °C and a mean annual precipitation of 577.8 mm. In contrast, Central Shaanxi Plain has a temperate monsoon climate with a mean annual temperature of 12.9 °C and a mean annual precipitation of 641.3 mm.

**Table 2:** Amounts of dry matter for the two crops used in this study.

Location		Latitude/longitude (°)	Mean annual daily maximum/minimum temperature(°C)	Sowing/harvest date	Amount of dry matter at harvest (kg/hm^2^)	Crop	Planting year	Reference
Central Shaanxi Plain	Fufeng	34.37/107.9	19.9/10.2	October 6/June 4	14 080	Winter wheat (xiaoyan22)	2006	X. Chen^42^
	Yangling	34.28/108.07	19.2/9.1	October 7/June 7	14 900		2002	H. Li^43^
				October 21/June 4	14 106		2009	Y.J. Chen^46^
				October 7/June 12	14 633		2010	Y.J. Chen^46^
				October 8/June 3	15 454		2007	M. Duan^44^
	Yangling	34.28/108.07	19.2/9.1	June 12/September 30	17 091	Corn (zhengdan958)	2009	M. Duan^44^
				June 16/October 10	14 175		2012	G.M. Jiang^45^
				June 16/October 13	14 873		2011	Y.J. Chen^46^
Northern Xinjiang	Shihezi	44.3/86.06	16.1/4.7	May 1/September 28	24 420	Corn (Xinyu8)	2001	B. Guo^47^
				April 28/October 1	35 845	Corn (liangyu66)	2013	B.Y. Guo^47^
				April 5/July 4	20 799	Spring wheat (xinchun6)	2013	J. Hu^49^
				March 28/July 4	17 377		2014	Y.B. Shen^50^
				March 25/July 1	18 671		2009	Y.W. Cheng^51^

## Results

### GDD from sowing to various developmental stages

**Figure 2:**
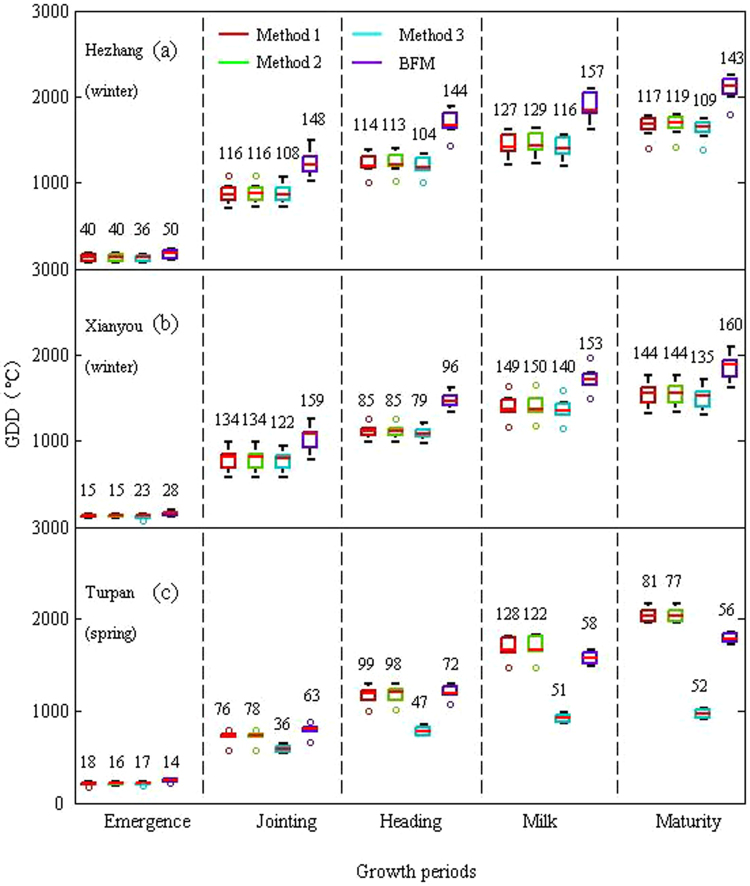
Box plots of GDD required to reach the developmental stages after sowing calculated by the four methods for wheat at three stations: Hezhang (**a**), Xianyou (**b**), and Turpan (**c**). Numbers indicate standard deviations.

**Figure 3:**
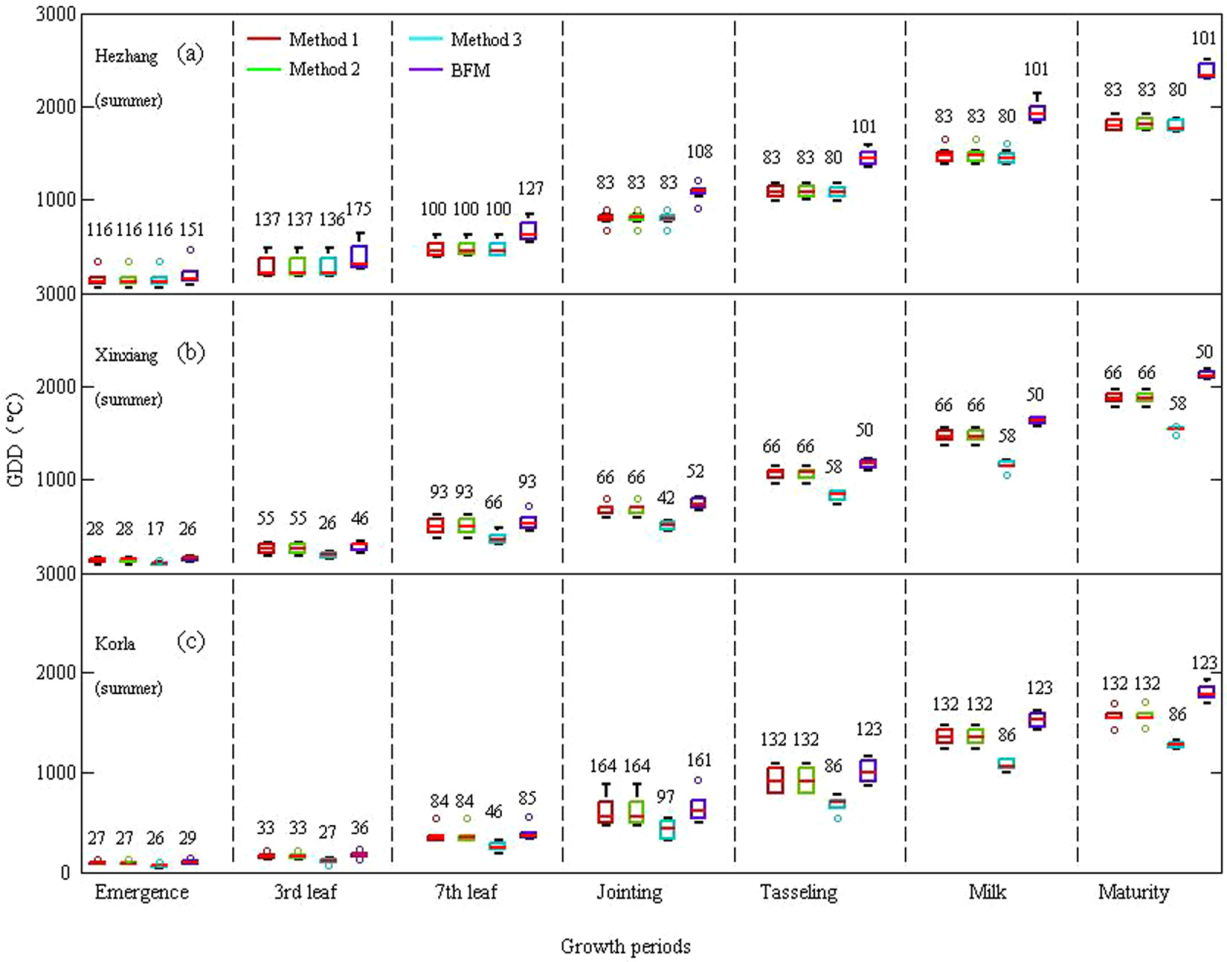
Box plots of GDD required to reach the developmental stages after sowing calculated by the four methods for corn at three stations: Hezhang (**a**), Xinxiang (**b**), and Korla (**c**). Numbers indicate standard deviations.

The GDD required to reach the various developmental stages calculated by the four methods for the two crops are shown in Figs 2 and 3.

GDD from sowing to a developmental stage could differ between the calculation methods, even for the same crop at the same station. For example, the GDD requirements from sowing to maturity for spring wheat at the Turpan station were 2167.85 ± 81, 2175.30 ± 77, 957.39 ± 52, and 1833.13 ± 56 °Cd for Method 1, Method 2, Method 3, and BFM, respectively. GDD from sowing to a particular developmental stage calculated by a particular method could differ between environments, even for the same crop. For example, the GDD requirement from sowing to maturity for winter wheat calculated by Method 1 was 1658.39 ± 117 °Cd for the Hezhang station and 1542.17 ± 144 °Cd for the Xianyou station. However, the GDD required from sowing to maturity for spring wheat calculated by Method 3 was much lower than that calculated by the other three methods, especially Methods 1 and 2 (the GDD calculated by Method 3 was half that calculated by Methods 1 and 2).

The standard deviations (SDs) of GDDs calculated by Methods 1–3 for winter wheat and corn were generally lower than those calculated by BFM, and the SDs were the lowest for Method 3. However, a lower SD did not necessarily indicate that one method was better than the others because the mean annual GDD calculated by the method may also be lower (e.g., Method 3). GDD has been used to describe crop development and the duration of a process or the time required to reach a particular stage, and confirming the merit of the four methods based only on GDD is thus difficult.

### CV for the prediction of developmental stage

#### CV for corn

The CVs of dates from sowing to the developmental stages predicted by the four methods were generally similar for corn (Table 3). The CVs calculated by Methods 1 and 2 were higher than those of Method 3 and BFM for the three locations. The CVs calculated by BFM were the lowest for two locations (Xinxiang and Hezhang). In contrast, the predictions by Method 3 were better from the 7th leaf stage to maturity in Korla than from emergence to the 3rd leaf stage compared with those of the other three methods. The new method (BFM) was better than Methods 1 and 2 for all cases. The CVs calculated by BFM were approximately 1 d (2%) lower in some cases (e.g., tasseling in Xinxiang and milk in Korla) relative to the other three methods. Methods 1 and 2 usually produced similar results.

**Table 3:** CV (%) of the predicted developmental stages for corn.

Location	Method	Emergence	3rd leaf	7th leaf	Jointing	Tasseling	Milk	Maturity
Xinxiang	Day after sowing	7.9	14.3	26.7	36.0	55.7	77.1	103.1
	Method 1	1.3 (17%)	2.7 (19%)	4.6 (17%)	3.3 (9%)	3.4 (6%)	3.4 (4%)	3.3 (3%)
	Method 2	1.3 (17%)	2.7 (19%)	4.6 (17%)	3.3 (9%)	3.4 (6%)	3.4 (4%)	3.3 (3%)
	Method 3	1 (13%)	1.7 (12%)	4.4 (16%)	2.8 (8%)	3.8 (7%)	3.5 (5%)	2.1 (2%)
	BFM	1.1 (14%)	2 (14%)	4.2 (16%)	2.4 (7%)	2.3 (4%)	1.9 (2%)	2.1 (2%)
Hezhang	Day after sowing	16.6	29.2	52.2	74.6	94.6	121.4	147.8
	Method 1	9.5 (57%)	12.4 (42%)	9.3 (18%)	7.2 (10%)	6.9 (7%)	8.9 (7%)	6.7 (5%)
	Method 2	9.5 (57%)	12.4 (42%)	9.3 (18%)	7.2 (10%)	6.9 (7%)	8.9 (7%)	6.7 (5%)
	Method 3	9.5 (57%)	12.3 (42%)	9.3 (18%)	7.3 (10%)	6.7 (7%)	8.1 (7%)	5.9 (4%)
	BFM	9.2 (55%)	11.6 (40%)	8.6 (16%)	7 (9%)	6.3 (7%)	8.3 (7%)	6.1 (4%)
Korla	Day after sowing	4.8	8.3	19.0	31.5	49.3	77.8	99.8
	Method 1	1.2 (25%)	1.5 (18%)	4 (21%)	8.2 (26%)	6.9 (14%)	5.1 (7%)	5.5 (6%)
	Method 2	1.2 (25%)	1.5 (18%)	4 (21%)	8.2 (26%)	6.9 (14%)	5.1 (7%)	5.6 (6%)
	Method 3	1.6 (33%)	1.8 (22%)	3.2 (17%)	6.9 (22%)	6.1 (12%)	4.1 (5%)	2.6 (3%)
	BFM	1.2 (25%)	1.5 (18%)	3.8 (20%)	7.5 (24%)	5.9 (12%)	4.1 (5%)	4.5 (5%)

Note: Figures in brackets are the percentages of the CV after sowing.

#### CV for spring wheat

For spring wheat, the CVs calculated by BFM for predicting the developmental stages since sowing were the lowest, followed by Method 3 and Methods 1 and 2 (see Turpan in Table 4). Method 2 was slightly better than Method 1. The advantage of BFM tended to increase; e.g., compared with Method 2, the CV of the jointing date predicted by BFM was 1 d lower and the CV of the milk date was 3.1 d lower. CV from sowing to heading was lower for Method 3 than that for the other methods, but the CVs of the method increased with development, especially by maturity (e.g., CV was 5.7 d for Method 3 and approximately 4 d for the other methods).

**Table 4:** CV (%) of the predicted developmental stages for wheat.

Location	Method	Emergence	Jointing	Heading	Milk	Maturity
Turpan	Days after sowing	24.3	55.5	75.0	95.5	108.7
	Method 1	2.1 (9%)	5.9 (11%)	6.4 (8%)	7.2 (7%)	4.2 (4%)
	Method 2	1.8 (7%)	6 (11%)	6.3 (8%)	7.1 (7%)	4.2 (4%)
	Method 3	1.9 (8%)	3.4 (6%)	4.5 (6%)	5.2 (5%)	5.7 (5%)
	BFM	2.0 (8%)	5.0 (9%)	5.0 (7%)	3.8 (4%)	3.2 (3%)
Hezhang	Days after sowing	9.6	122.2	157.4	186.0	209.3
	Method 1	2.9 (30%)	16.4 (13%)	14.6 (9%)	16.5 (9%)	14.7 (7%)
	Method 2	2.9 (30%)	16.2 (13%)	14.4 (9%)	16.5 (9%)	14.9 (7%)
	Method 3	2.6 (27%)	15.3 (13%)	13.6 (9%)	15.4 (8%)	14.1 (7%)
	BFM	2.7 (28%)	14.9 (12%)	13.3 (8%)	15.3 (8%)	14 (7%)
Xianyou	Days after sowing	8.0	58.6	84.7	125.4	140.9
	Method 1	0.9 (11%)	10.1 (17%)	6.4 (8%)	13.4 (11%)	13.2 (9%)
	Method 2	0.9 (11%)	10.1 (17%)	6.4 (8%)	13.4 (11%)	13.1 (9%)
	Method 3	1.5 (19%)	9.3 (16%)	6.2 (7%)	12.8 (10%)	12.6 (9%)
	BFM	1.4 (18%)	9.1 (16%)	5.5 (6%)	11 (9%)	11.9 (8%)

Note: Figures in brackets are the percentages of the CV after sowing.

#### CV for winter wheat

For winter wheat, the CVs were the lowest for BFM for predicting the developmental stages since sowing, followed by Method 3 and Methods 1 and 2; however, the advantages of BFM and Method 3 were not larger than those for spring wheat (see Hezhang and Xianyou in Table 4). Methods 1 and 2 did not differ in most cases, as expected. CVs calculated by Method 3 were usually nearly 0.5 d lower than those of Methods 1 and 2. In contrast, CVs calculated by BFM were usually <1 d.

### The performance of the four methods for predicting developmental stage

We further analyzed the performance of the four methods for predicting developmental stage using a refined index of agreement, d_r_, which is widely used as a goodness-of-fit indicator (Table 5). All methods performed well for all locations. For winter wheat, d_r_ for BFM and Method 3 was similar in two locations, and the two methods were generally better than Methods 1 and 2. BFM was better than the other three methods for spring wheat in Turpan, followed by Methods 1 and 2 and Method 3. There was no difference among Methods 1, 2 and 3 in three locations for corn, and BFM had better performance than the three methods.

**Table 5:** D_r_ for the prediction of developmental stage using the four methods.

Crop	Location	Year	Method 1	Method 2	Method 3	BFM
Spring wheat	Turpan	2005	0.97	0.97	0.98	0.99
		2006	0.86	0.86	0.90	0.90
		2007	0.93	0.94	0.92	0.96
Winter wheat	Hezhang	2007–2008	0.97	0.96	0.97	0.97
		2009–2010	0.95	0.94	0.95	0.95
		2010–2011	0.91	0.92	0.91	0.93
	Xianyou	2006–2007	0.84	0.84	0.85	0.86
		2008–2009	0.93	0.93	0.94	0.94
		2009–2010	0.92	0.92	0.94	0.94
Summer corn	Korla	2011	0.94	0.95	0.94	0.96
		2012	0.95	0.95	0.93	0.96
		2013	0.96	0.96	0.97	0.97
	Hezhang	2005	0.94	0.94	0.93	0.94
		2008	0.96	0.96	0.97	0.95
		2009	0.94	0.94	0.93	0.95
	Xinxiang	2011	0.88	0.88	0.88	0.96
		2012	0.96	0.96	0.95	0.98
		2013	0.90	0.90	0.91	0.93

### The performance of describing the accumulation of dry matter

**Figure 4:**
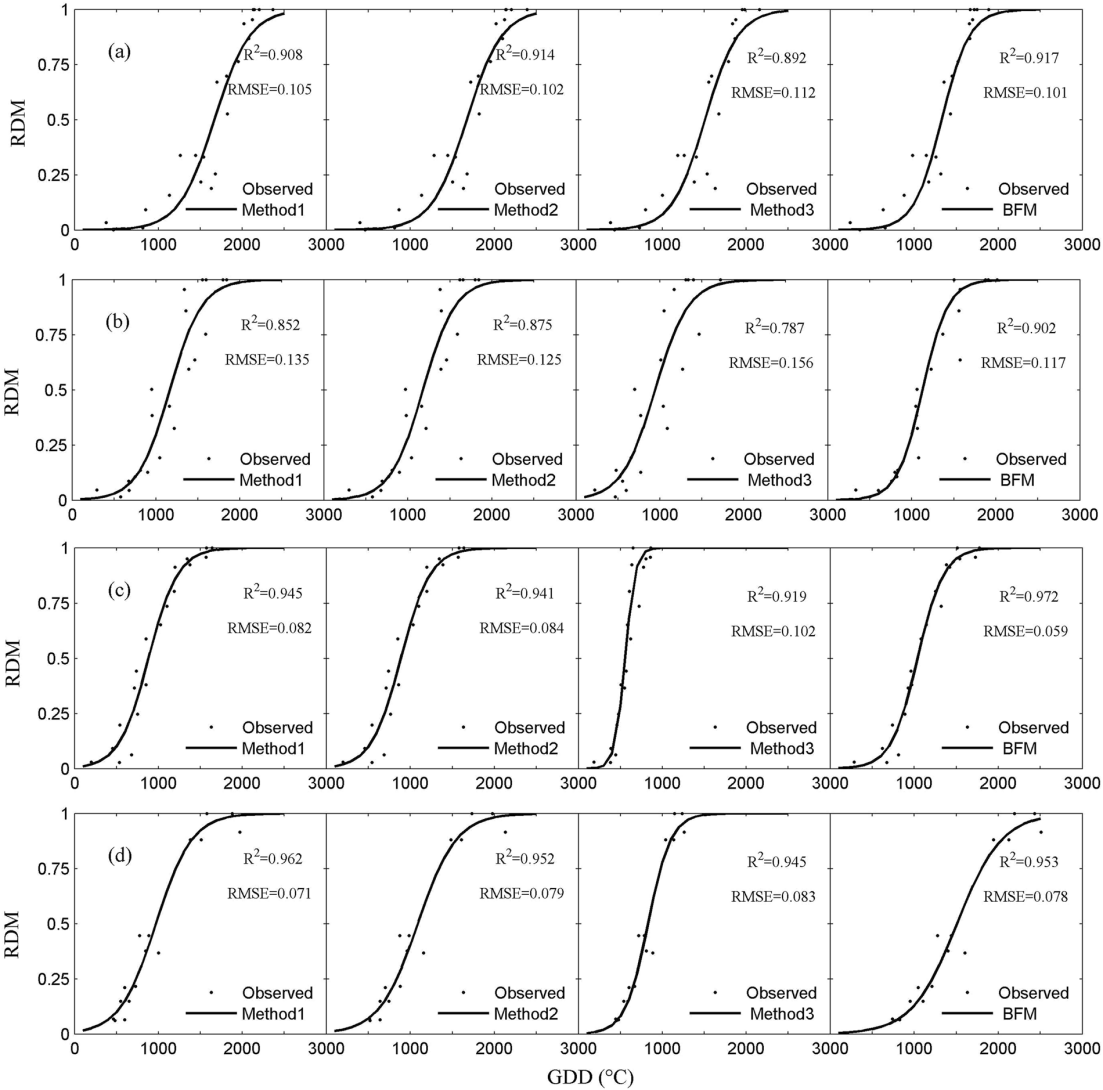
Relative accumulation of dry matter (RDM) fitted with a normalized logistic model as a function of GDD. Northern Xinjiang (**a**) and central Shaanxi Plain (**b**) for corn; northern Xinjiang for spring wheat (**c**); central Shaanxi Plain for winter wheat (**d**).

The accumulation of dry matter was described well with the normalized logistic model as a function of GDD calculated by the four methods for the two locations (Fig. 4), with an RMSE between observed and fitted values by the four methods within 0.11 for wheat and 0.16 for corn. The methods agreed the most for northern Xinjiang. RMSE fitted with GDD calculated by BFM was the smallest, especially for spring wheat (RMSE was 0.06 for BFM and >0.08 for the other methods), as expected. Methods 1 and 2 did not generally differ for the two crops and the two locations. Method 3 was not better than Methods 1 or 2 in most cases. The curve for the accumulation of dry matter was steeper for Method 3 than the other methods because GDD was lower for Method 3 in some cases (Fig. 4c,d).

## Discussion

Most of the methods for calculating GDD assume that crop development responds linearly to temperature. This calculation of GDD is appropriate for predicting plant development if several conditions are met^9,24^. The use of GDD to describe the duration of growth is reasonable when plant developmental rates for a region are linear over a wide range of temperatures^52^. However, several experiments have indicated that the rates could also respond nonlinearly to temperature^24^. To our knowledge, crop plants do not survive the high temperatures that could stop development, and interest in the production of crops is highest when environmental temperatures are near T_opt_. However, Eqs (5) and (6) indicate that high temperature remains the largest contributor to crop development and they are thus obviously inappropriate. Temperatures above T_opt_ will retard crop development.

### Conditions applicable to the GDD calculation methods

The concept of degree days, with its linear relationship between temperature and developmental rate, is inadequate for simulating field populations under highly variable temperatures^8^. The methods of an optimized developmental response (OR) to temperature (e.g., Method 3 and BFM) are less convenient than the methods of a linear developmental response (LR) (e.g., Methods 1 and 2) for calculating GDD. However, the OR methods generally have higher predictive accuracy, which is also supported by the physiological interpretations of optimal, supraoptimal, and limiting temperatures^11^.

OR methods should thus have an advantage over LR methods. The OR methods were more accurate than both LR methods for predicting the dates of the developmental stages for corn, BFM was slightly better than Method 3 in some cases, and Methods 1 and 2 were slightly worse than Method 3. BFM best predicted the dates of the developmental stages for wheat since sowing, especially for spring wheat.

**Figure 5:**
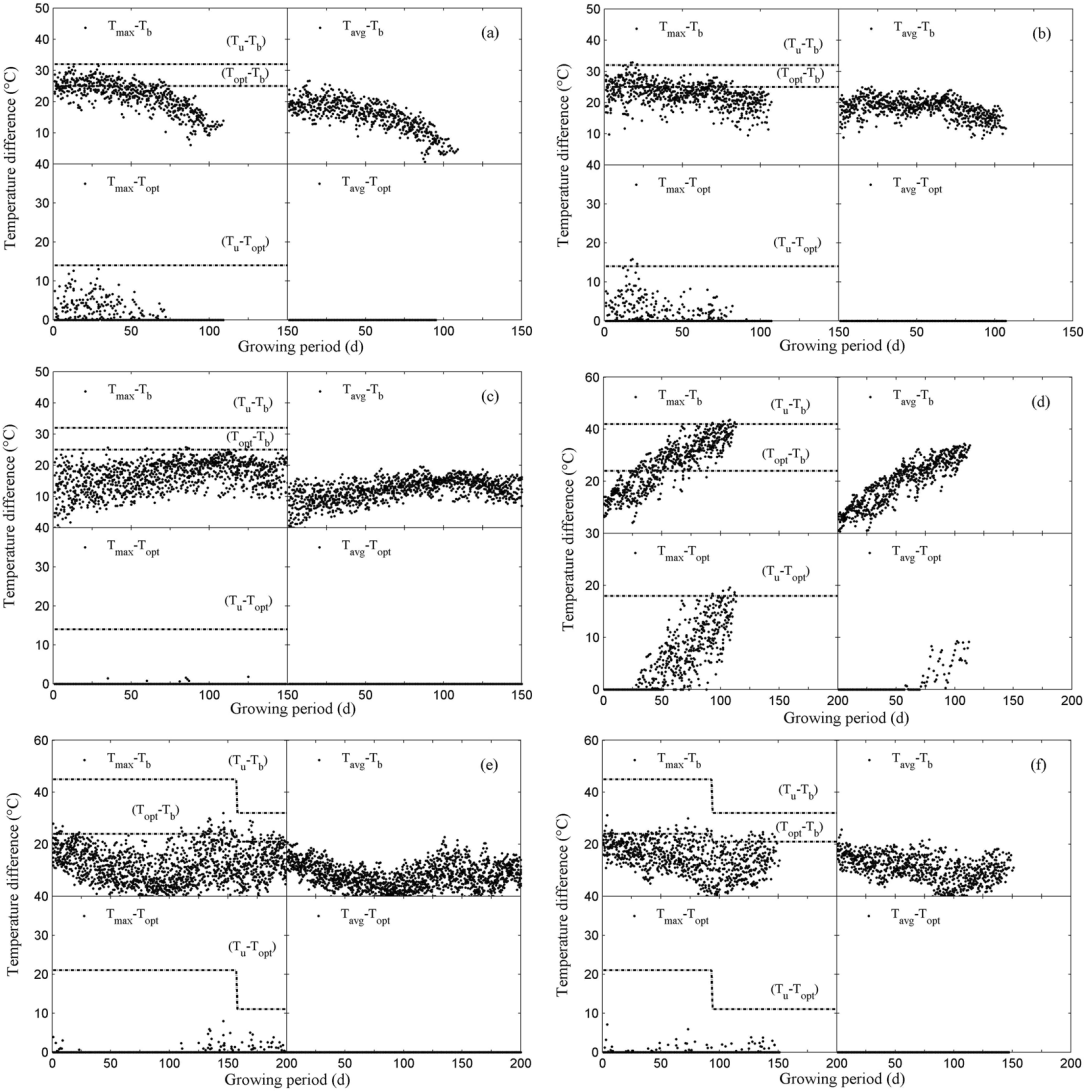
Temperature differences after sowing during the growing season. Korla (**a**), Xinxiang (**b**), and Hezhang (**c**) for corn; Turpan (**d**) for spring wheat; Hezhang (**e**) and Xianyou (**f**) for winter wheat. T_max_, maximum temperature; T_avg_, average temperature; T_u_, upper threshold temperature; T_opt_, optimum temperature; T_b_, lower threshold temperature. T_max_ − T_b_ is the temperature difference between T_max_ and T_b_, T_max_ − T_opt_ is the temperature difference between T_max_ and T_opt_, T_avg_ − T_b_ is the temperature difference between T_avg_ and T_b_, T_avg_ − T_opt_ is the temperature difference between T_avg_ and T_opt_, T_u_ − T_b_ is the temperature difference between T_u_ and T_b_, T_opt_ − T_b_ is the temperature difference between T_opt_ and T_b_, and T_u_ − T_opt_ is the temperature difference between T_u_ and T_opt_.

T_opt_ is usually well above 30 °C for corn, and T_max_ is lower or slightly higher than usual for most growing stages. T_opt_ is lower for wheat than that for corn, but the growth period of winter wheat is generally from mid-October to the end of May; in addition, T_max_ is usually lower than T_opt_ because temperatures in winter and spring are usually low. The OR methods would have more advantages with more days when temperatures are above T_opt_ throughout the crop growth period. The temperature differences from sowing to the developmental stages for the two crops were thus used for further analysis (Fig. 5).

Three locations (Korla, Xinxiang, and Hezhang) for corn had only a few days with T_avg_ > T_opt_ but T_m_ > T_opt_ for more than one third of the days at Korla and Xinxiang from sowing to the 7th leaf stage and for approximately 5–20% of the days during the other developmental stages. However, a few days at Hezhang had T_max_ > T_opt_. This analysis demonstrated that the CVs calculated by the four methods were similar for Hezhang but CVs calculated by BFM were lower than those of the other methods for two other locations (Korla and Xinxiang). Figure 5 further illustrates why BFM was more precise at predicting developmental dates than the other methods for wheat, especially for spring wheat in Turpan.

The function of ORs to temperature has a large influence on the performance of the OR methods. Method 3 was superior to both LR methods (Methods 1 and 2) in most cases and opposite in some cases. For example, the CVs from sowing to milk and maturity for spring wheat at Turpan were higher for Method 3 than both LR methods, and RMSE for the accumulation of dry matter predicted by Method 3 was 0.1 for spring wheat for Northern Xinjiang. In contrast, BFM was more stable and precise than Method 3.

### Sensitivity analysis of the cardinal temperatures in the methods for calculating GDD

The three cardinal temperatures (T_b_, T_opt_, and T_u_) can differ between varieties of a particular crop^53^. Small changes in the cardinal temperatures could affect the accuracy of the methods in predicting developmental stages. A sensitivity analysis was used to determine the uncertainty of the effect of the three cardinal temperatures on the precision of the four methods.

**Figure 6:**
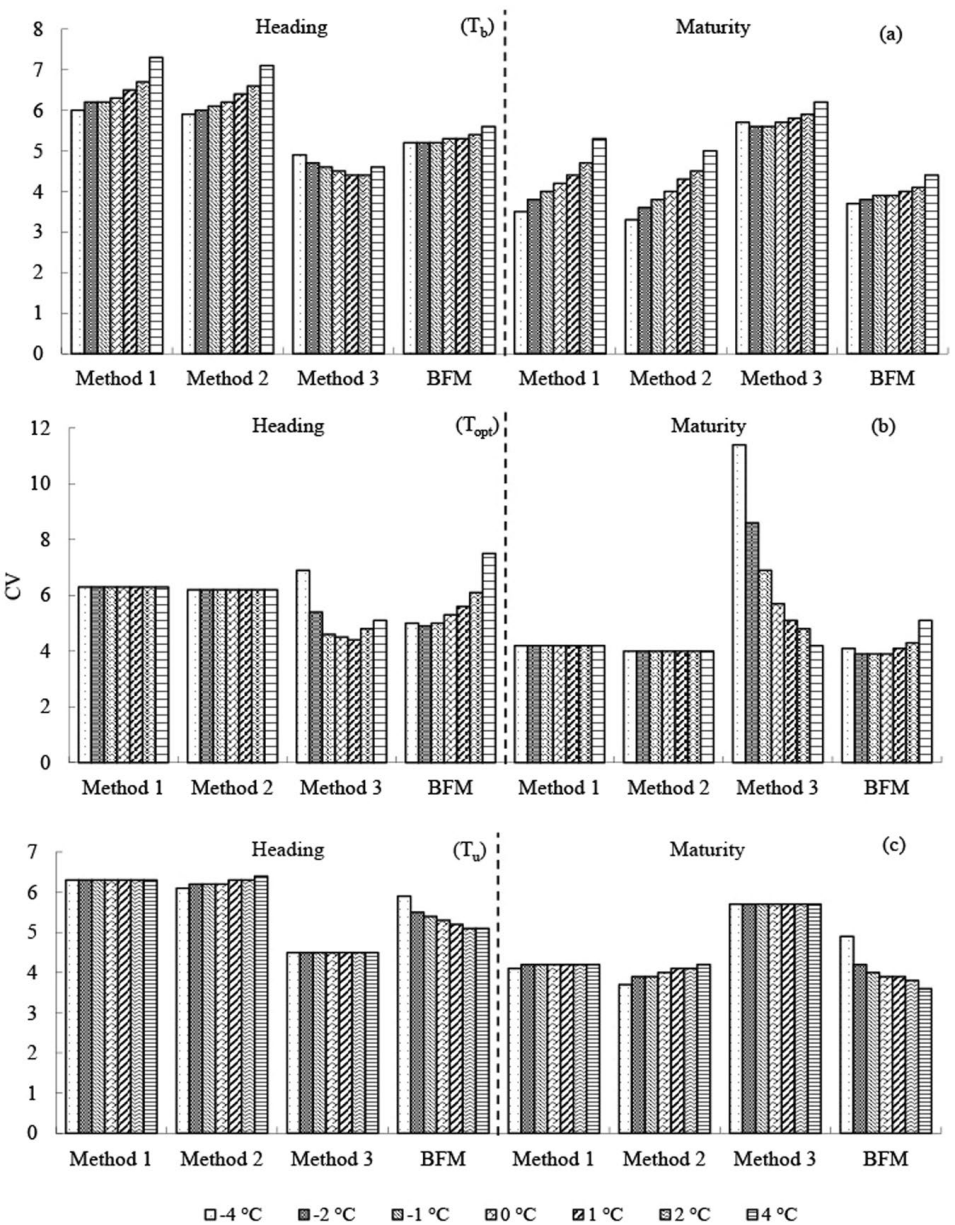
Uncertainty of the effect of the three cardinal temperatures on the precision of the four methods for spring wheat in Turpan.

**Figure 7:**
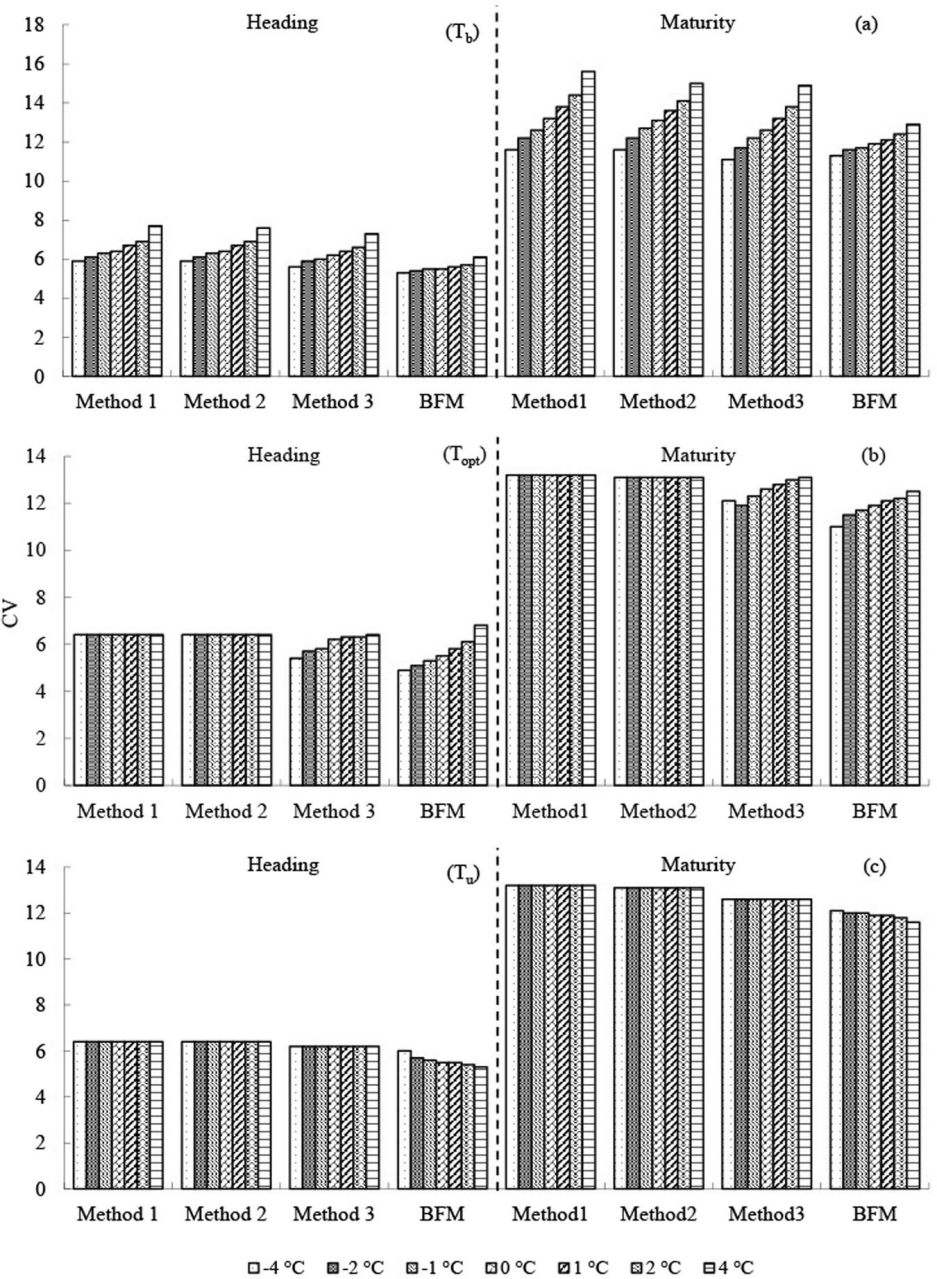
Uncertainty of the effect of the three cardinal temperatures on the precision of the four methods for winter wheat in Xianyou.

**Figure 8:**
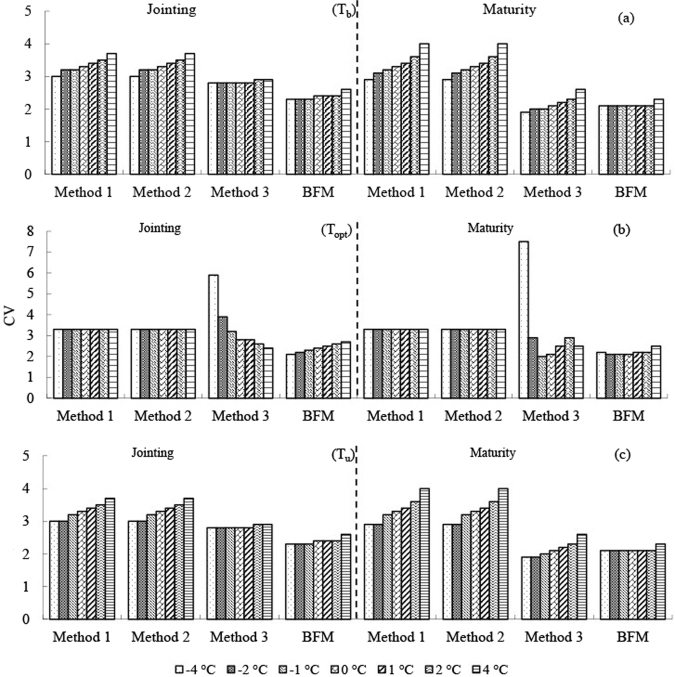
Uncertainty of the effect of the three cardinal temperatures on the precision of the four methods for corn in Xinxiang.

One of the three cardinal temperatures changed by −4, −2, −1, 1, 2, and 4 °C, and the other two cardinal temperatures remained unchanged. The CVs of two predicted developmental stages (heading and maturity for wheat, jointing and maturity for corn) since sowing at three stations (Turpan for spring wheat, Xianyou for winter wheat, and Xinxiang for corn) were used for the analysis (Figs 6–8).

### T_b_ sensitivity

A change of T_b_ from −4 to 4 °C increased CVs of the predicted developmental stages for the two crops for Methods 1 and 2; Method 3 was similar to Methods 1 and 2, and the CVs of Method 3 decreased in some cases. In contrast, a change of T_b_ from −4 to 4 °C slightly increased the CVs of the predicted developmental stages for BFM. Sensitivity to T_b_ was highest for Methods 1 and 2, followed by Method 3 and was generally lowest for BFM.

### T_opt_ sensitivity

Only Method 3 and BFM are discussed in this section because T_opt_ was not used for calculating GDD by Methods 1 or 2. Changes of T_opt_ led to highly variable precision for Method 3 in some cases (e.g., CVs doubled when predicting the maturity stage for spring wheat in Turpan and corn in Xinxiang) (Figs 6 and 8). In contrast, changes of T_opt_ only slightly increased the CVs of the predicted developmental stages for BFM, which generally remained the most precise of the four methods.

### T_u_ sensitivity

Changes of T_u_ had little effect on Methods 1–3 for the two crops, as expected, because the environmental temperatures in most of the growth periods were usually <T_u_. An increase in T_u_ could improve the precision of predicting the developmental stages for wheat for BFM to some extent, especially for spring wheat in hot environments (e.g., Turpan).

## Conclusions

Methods 1 and 2 were generally appropriate if T_max_ < T_opt_ for the crop. Method 2 performed as well as Method 1 in most cases but Method 2 was generally slightly better.

For both crops, Method 3 and BFM generally performed better than Methods 1 and 2 at predicting the developmental stages since sowing and predicting the accumulation of dry matter with the normalized logistic model as a function of GDD. However, the stability of Method 3 was unsatisfactory because changes in the cardinal temperatures led to highly variable precision in some cases. BFM was sufficiently stable and more precise than the other methods for T_opt_ < T_max_. This study used specific examples but the results can be applied to any environment or crop.
